# Garlic-Extract-Functionalized Fe_3_O_4_ Magnetic Colloids as Building Blocks for Gel-like Emulsified-Oil Capture

**DOI:** 10.3390/gels12080723

**Published:** 2026-08-14

**Authors:** Wanxin Hao, Yan Wu, Mengting Zhang, Yunpeng Fan, Gang Yan, Shouyu Zhao

**Affiliations:** 1 Hubei Key Laboratory of Petroleum Geochemistry and Environment, Yangtze University, Wuhan 430100, China; haowanxin@foxmail.com (W.H.); q7656467@163.com (Y.W.); mengtingzhang0207@foxmail.com (M.Z.); 2 Key Laboratory of Exploration Technologies for Oil and Gas Resources of Ministry of Education, Yangtze University, Wuhan 430100, China; 3 Oil & Gas Technology Research Institute, PetroChina Changqing Oilfield Company, Xi’an 710016, China; fyp31_cq@petrochina.com.cn; 4 Mud Logging Company, Bohai Drilling Engineering Company Limited, China National Petroleum Corporation, Tianjin 300280, China; 15971598632@163.com

**Keywords:** magnetic colloids, gel-like colloidal assembly, organic–inorganic hybrid, garlic extract, organosulfur compounds, emulsified oil, magnetic separation

## Abstract

Stable emulsified oil droplets are difficult to separate because of persistent interfacial films and colloidal stability. Garlic-extract-functionalized Fe_3_O_4_ magnetic colloids, operationally denoted Allicin@Fe_3_O_4_, were prepared by in situ coprecipitation as organic–inorganic building blocks for gel-like interfacial capture. The sample name identifies the allicin-containing garlic-extract route and does not imply that allicin was proven to be the predominant surface species. SEM, FTIR, and XRD supported deposition of an organic, sulfur/oxygen-containing surface layer and retention of crystalline Fe_3_O_4_, but these methods are not species-specific. The colloids removed more than 95% of emulsified oil within 20 min and reached an experimental equilibrium apparent uptake of approximately 380 mg·g^−1^. Tests across pH 3–11, 0–50 g·L^−1^ NaCl, 15–55 °C, and representative coexisting ions showed robust but condition-dependent removal. Removal remained above 90% after five reuse cycles and was approximately 84% after ten cycles. Calculations using allicin as a representative garlic organosulfur molecule suggest how polar sulfur/oxygen regions and allyl segments could favor oil–droplet anchoring and association; they do not establish the surface composition of the extract-derived coating. The capture behavior is consistent with transient, gel-like particle–droplet association coupled to magnetic recovery. These results connect bio-derived surface functionalization, gel-related colloidal structuring, and magnetic separation under the tested batch conditions.

## 1. Introduction

Oily wastewater generated during petroleum extraction, refining, and petrochemical processing is difficult to treat because dispersed and emulsified oil droplets behave as stable colloidal systems rather than readily separable bulk oil phases [1,2,3,4]. Natural surface-active components, added surfactants, and dissolved organic matter can reinforce oil–water interfacial films, inhibit droplet coalescence, and sustain small droplets in the aqueous phase. These effects contribute to poor biodegradability and persistent environmental risk when oily effluents are discharged without sufficient treatment [5,6]. Materials that can regulate the oil–water interface while remaining recoverable are therefore needed for efficient treatment under chemically complex conditions [7].

Colloidal gels are generally associated with interaction-driven particle networks whose structural arrest gives the dispersion solid-like or gel-like behavior. In emulsified systems, interfacially active particles may attach to several droplets, promote droplet association, and generate transient particle–droplet assemblies before macroscopic phase separation. This concept provides a useful bridge between conventional adsorption and gel science: the dispersed particles are considered not merely as isolated adsorption sites, but as building blocks that reorganize the multiphase interface. In the present work, the term “gel-like interfacial assembly” is used conservatively for transient, interaction-driven particle-droplet association during capture; it does not denote a chemically crosslinked polymer gel or claim rheologically confirmed bulk gelation.

Magnetic nanomaterials, especially Fe_3_O_4_, are attractive building blocks for such interfacial assemblies because they combine tunable surface chemistry with rapid recovery under an external magnetic field [8]. However, pristine Fe_3_O_4_ nanoparticles are susceptible to magnetic agglomeration and often show limited affinity toward surfactant-stabilized oil droplets [9,10]. Surface functionalization is thus required to balance aqueous dispersibility, oil–water interfacial affinity, and magnetic collectability. Properly designed surface layers can convert Fe_3_O_4_ from a passive magnetic solid into an interfacially active colloidal building block that promotes oil–droplet attachment, association, and separation [11,12].

This design problem is also relevant to current gel-material research, which increasingly includes organic–inorganic hybrids, nanoparticle assemblies, stimuli-responsive soft materials, and environmentally oriented water-purification systems. A magnetically recoverable, gel-like colloidal assembly could couple interfacial structuring with field-assisted collection without requiring a permanent polymer network. For oily wastewater treatment, the key requirements are a surface chemistry that recognizes heterogeneous oil–droplet interfaces, sufficient colloidal association to destabilize the emulsion, tolerance to pH and ionic-strength changes, and repeated magnetic recovery. Bio-derived functional molecules offer a potential route to combining these functions while reducing reliance on fully synthetic modifiers.

Allicin, a naturally occurring thiosulfinate compound from garlic, contains sulfur- and oxygen-containing moieties such as –S–S– and –S(O)– together with allyl segments that contribute to molecular polarity and weak hydrophobic interactions [13,14]. This anisotropic chemical structure is potentially suitable for a hybrid particle surface: the sulfur/oxygen region can interact with water and polar oil components, whereas the allyl segments can favor association with the oil phase. Although allicin and related garlic-derived components have been explored in biological and functional-material contexts [15], their use in magnetic colloidal building blocks for emulsified-oil capture remains limited, and the connection between molecular interaction, particle–droplet association, and magnetic separation has not been fully clarified [16].

In this work, an allicin-containing garlic extract was introduced during the in situ preparation of the magnetic colloids. The product is operationally denoted Allicin@Fe_3_O_4_ for continuity, but the garlic extract is chemically complex and may contain allicin, its transformation products, and other organosulfur and organic constituents. The notation therefore does not assert that allicin is the predominant surface species. We hypothesized that the bio-derived surface layer would enhance interfacial anchoring and promote transient gel-like particle–droplet association, while the Fe_3_O_4_ core would enable external-field recovery. The material was evaluated in terms of rapid emulsified-oil capture, concentration-dependent uptake, environmental robustness, and cyclic magnetic separation. Electrostatic-potential, frontier-orbital, and van der Waals-potential analyses of allicin were used only as a representative molecular model for a plausible organosulfur interaction motif. By connecting extract-derived surface functionalization, gel-related colloidal structuring, and magnetic recovery, this study aims to position the material as an organic–inorganic platform for emulsified oil–water purification while retaining the boundaries of the tested batch system.

## 2. Results and Discussion

### 2.1. Structural and Surface Features Relevant to Gel-like Colloidal Assembly

**Figure 1:**
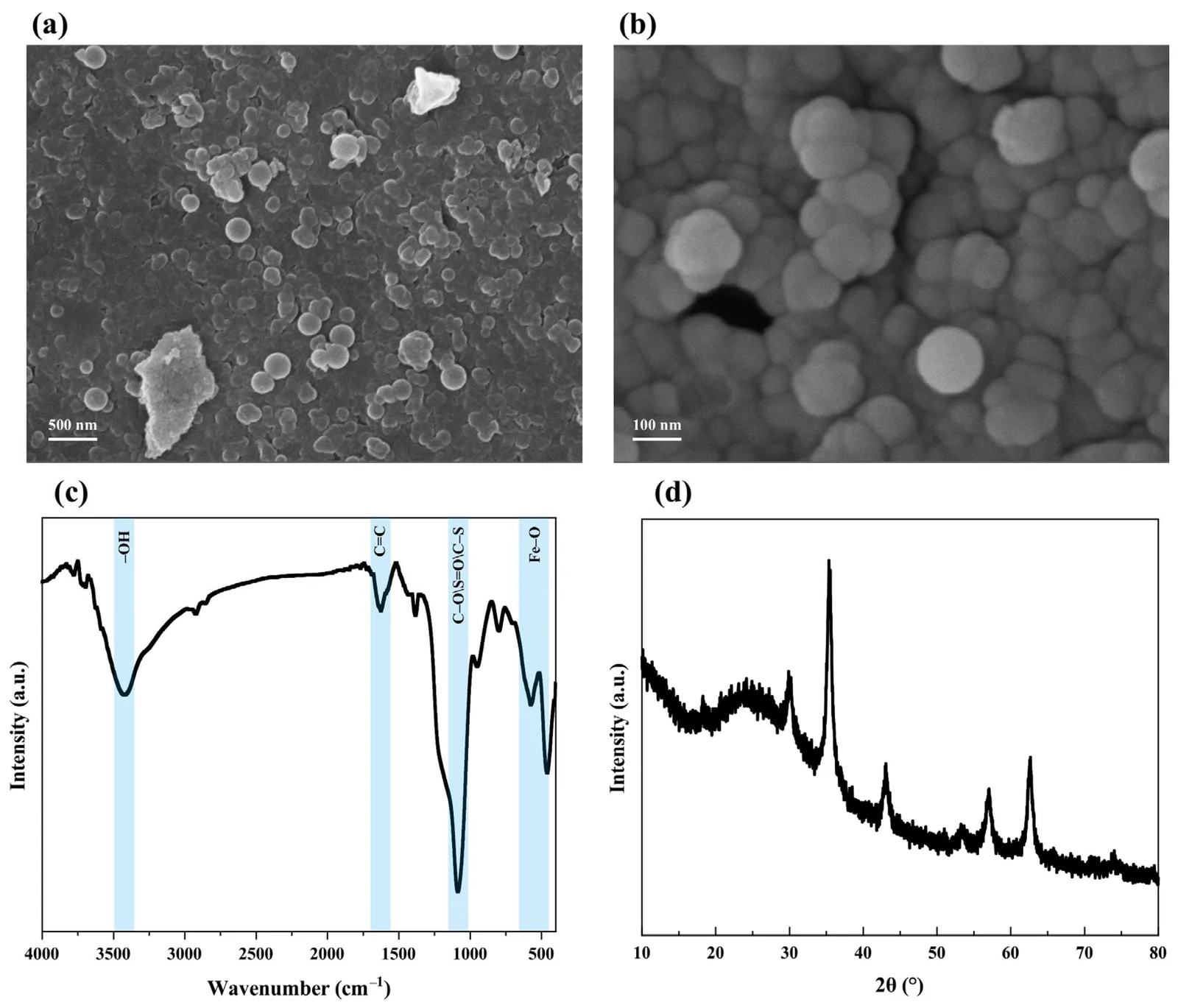
Morphological and structural characterization of Allicin@Fe_3_O_4_ nanoparticles by SEM (**a**,**b**), FTIR (**c**), and XRD (**d**).

The morphological and structural characteristics of the prepared Allicin@Fe_3_O_4_ composite are shown in Figure 1. The low-magnification SEM image (Figure 1a) shows approximately spherical primary particles together with clustered domains in the submicron range. In the high-magnification image (Figure 1b), the visible primary particles are nanoscale, with many apparent diameters on the order of approximately 50–150 nm, while larger features arise from aggregation. This range is an image-based estimate rather than a statistically representative particle-size distribution because TEM/DLS measurements were not performed. The dry-state morphology is consistent with partial magnetic or drying-induced association rather than complete particle isolation; the extract-derived surface layer may nevertheless moderate direct Fe_3_O_4_ contact and contribute to colloidal accessibility [9]. These images establish the particulate building blocks but do not, by themselves, demonstrate aqueous dispersion stability or bulk gelation [17].

FTIR was used to assess the surface chemical environment of Allicin@Fe_3_O_4_ (Figure 1c). The broad band near 3400 cm^−1^ is assigned to O–H stretching from Fe_3_O_4_ surface hydroxyls and adsorbed water [18]. Bands near 2920 and 2850 cm^−1^ indicate aliphatic C–H groups, and the 1040–1100 cm^−1^ region is consistent with overlapping C–O/S–O-containing functionalities in the extract-derived organic layer [19]. The strong band near 580 cm^−1^ is assigned to Fe–O vibration [20,21]. Because C–H and sulfur/oxygen bands are shared by multiple garlic-derived compounds and allicin is chemically labile, FTIR supports deposition of an organic sulfur/oxygen-containing layer but cannot identify allicin uniquely or establish it as the predominant surface species.

The XRD pattern (Figure 1d) shows diffraction peaks at 2θ = 30.2°, 35.5°, 43.2°, 53.5°, 57.1°, and 62.7°, corresponding to the (220), (311), (400), (422), (511), and (440) planes of Fe_3_O_4_ (JCPDS No. 19-0629), respectively. The absence of additional sharp peaks attributable to a crystalline organic phase is consistent with an amorphous or molecularly dispersed extract-derived surface layer; it does not identify the individual organic species. Thus, XRD confirms retention of the Fe_3_O_4_ crystalline phase, whereas the chemical identity and relative abundance of surface organosulfur compounds would require species-specific analysis, such as chromatographic or mass-spectrometric characterization [22].

Together, SEM, FTIR, and XRD support an organic–inorganic magnetic-colloid system with a crystalline Fe_3_O_4_ core and an extract-derived surface layer containing aliphatic and sulfur/oxygen functionalities. These features provide structural prerequisites for interfacial oil–droplet association and magnetic separation [23]. The available characterization does not prove allicin predominance, a quantitative particle-size distribution, aqueous colloidal stability, or rheological gelation; these points are now treated as limitations rather than inferred properties.

### 2.2. Rapid Interfacial Capture and Gel-like Particle–Droplet Association

**Figure 2:**
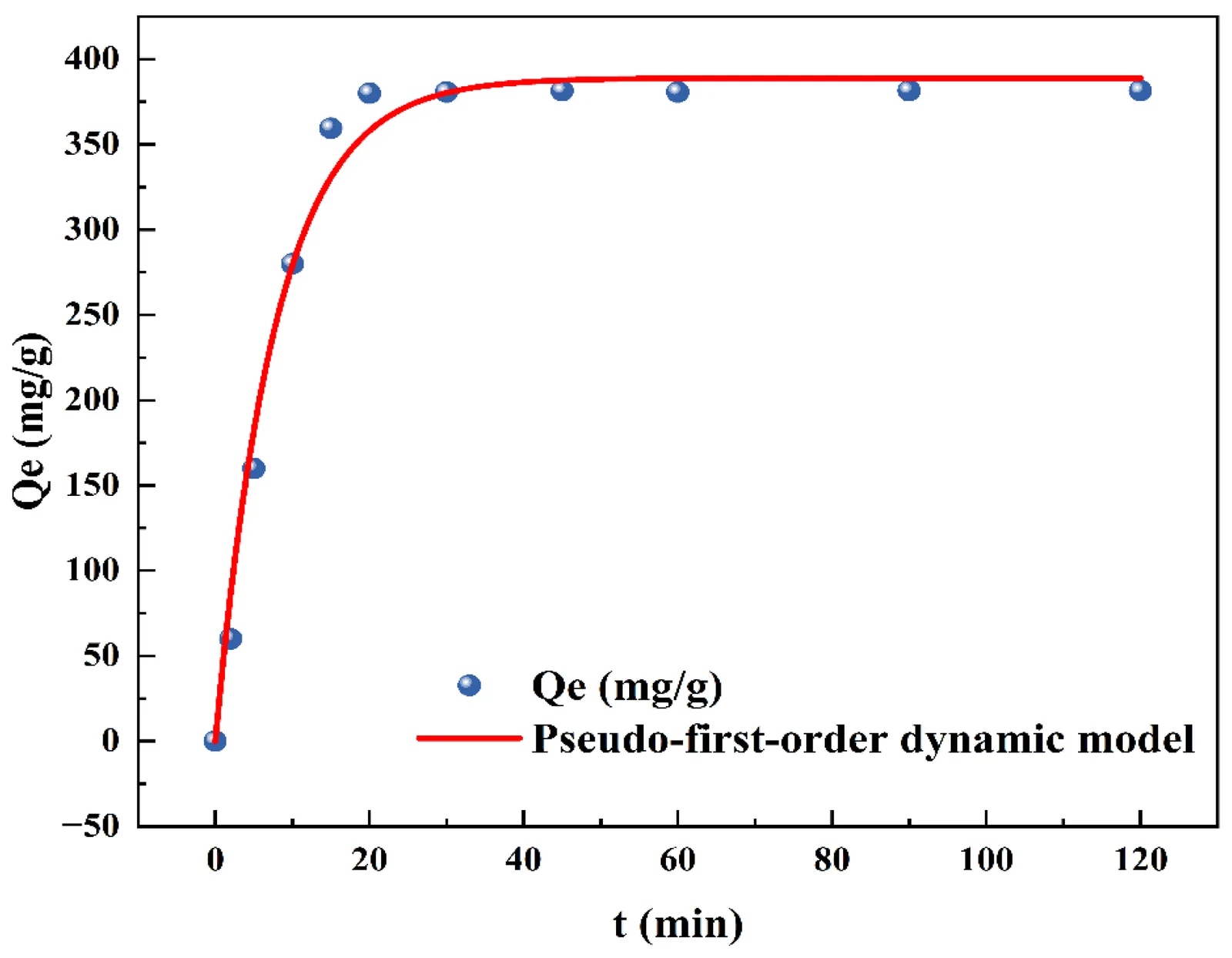
Oil–droplet capture kinetics of Allicin@Fe_3_O_4_ and fitting with the pseudo-first-order model.

As shown in Figure 2, the time-dependent removal of emulsified oil by Allicin@Fe_3_O_4_ can be divided into a rapid capture stage (0–20 min) and a subsequent slow equilibration stage (20–120 min). During the initial stage, the apparent uptake per unit mass increased sharply and reached more than 90% of the equilibrium value within approximately 20 min. This rapid response indicates that the allicin-functionalized magnetic colloids have a strong affinity for oil–droplet interfaces and low apparent mass-transfer resistance, enabling efficient contact, anchoring, and coalescence of emulsified oil droplets.

The equilibrium capacity stabilized at approximately 380–390 mg·g^−1^. The pseudo-first-order model fitted the experimental data well, suggesting that the rate of removal is closely related to the availability of active interfacial sites on the particle surface. In the present system, this model is used as a descriptive kinetic tool rather than as evidence for a purely conventional adsorption process. The actual removal pathway likely involves interfacial adhesion, oil–droplet aggregation, and magnetic colloid-assisted separation [24].

The rapid capture behavior is consistent with the material structure. Surface hydroxyl groups and sulfur/oxygen-containing constituents in the garlic-derived layer may interact with polar components at emulsified oil interfaces through hydrogen bonding, electrostatic interactions, and induced non-covalent interactions [11,25,26]. The magnetic Fe_3_O_4_ core provides a recoverable framework that facilitates solid–liquid separation after droplet capture [27,28,29]. Therefore, the kinetic results support the role of Allicin@Fe_3_O_4_ as an interfacially active magnetic colloid rather than only as a passive adsorbent. The rapid association is consistent with transient gel-like particle–droplet assemblies, although the kinetic fit alone is not evidence of rheological gelation.

### 2.3. Initial-Concentration Response and Apparent Oil–Droplet Capture Capacity

**Figure 3:**
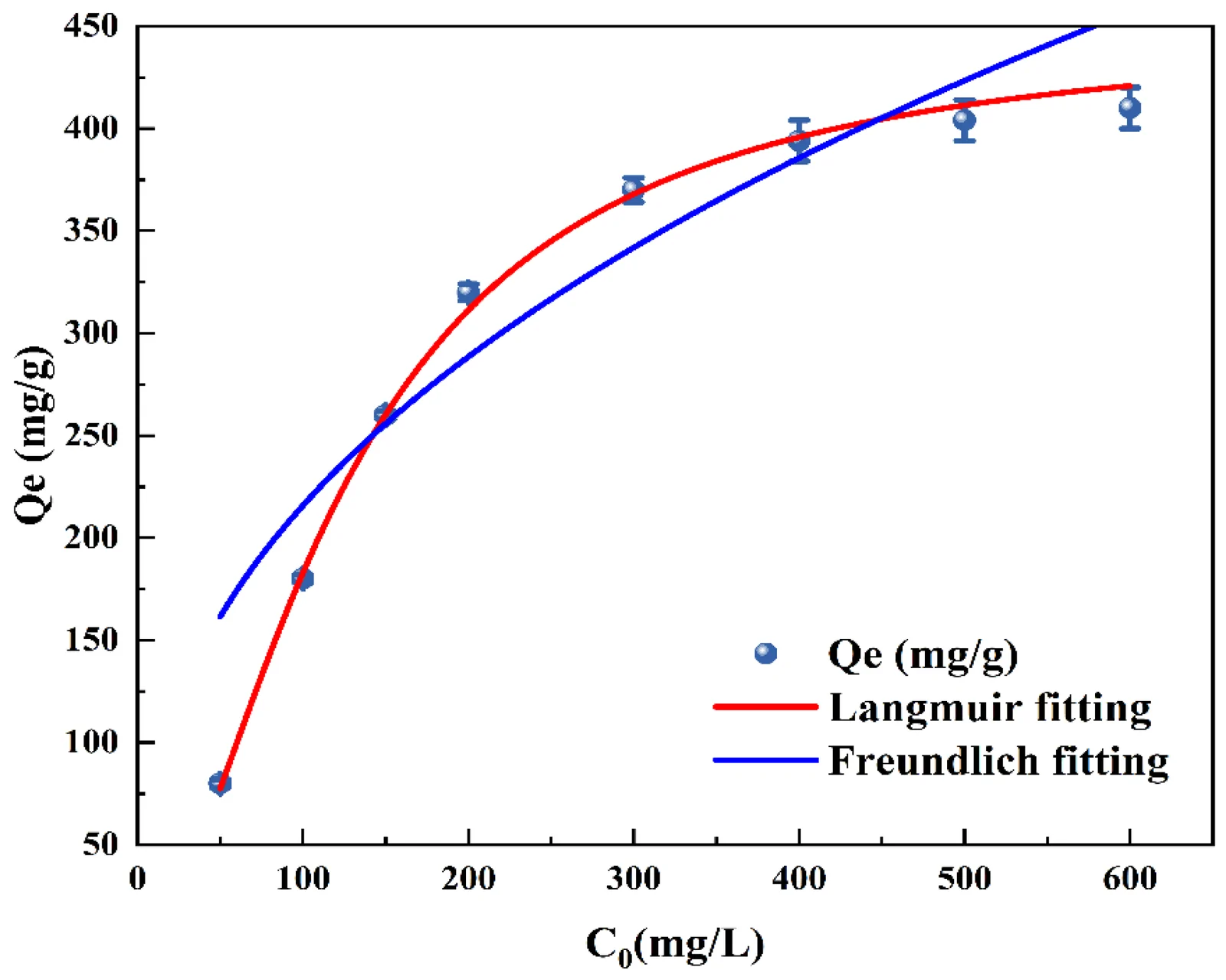
Initial concentration response of emulsified oil capture by Allicin@Fe_3_O_4_. Langmuir- and Freundlich-type curves are shown as empirical saturation functions and are not interpreted as strict equilibrium adsorption isotherms. Data points represent means from three independent batch experiments; error bars show standard deviation where visible (*n* = 3).

Figure 3 presents the initial concentration response of Allicin@Fe_3_O_4_ toward emulsified oil droplets. As the initial oil concentration (C0) increased from 50 to 600 mg·L^−1^, the equilibrium apparent uptake increased from approximately 80 to more than 400 mg·g^−1^. The higher initial concentration increases the frequency of colloid–droplet encounters and the mass of oil available for capture. Because the horizontal axis is C0 rather than the equilibrium aqueous concentration, these data are not presented as a strict adsorption isotherm.

Langmuir- and Freundlich-type curves are retained in Figure 3 only as empirical saturation functions for descriptive comparison with prior magnetic particle literature [11]. They are not interpreted as evidence of equilibrium molecular adsorption or an ideal monolayer. A rigorous adsorption isotherm would require equilibrium aqueous concentrations on the horizontal axis and adsorbed mass per unit sorbent mass on the vertical axis. In this emulsion system, the reported quantity is therefore termed apparent capture capacity and reflects the combined contributions of colloidal collision, interfacial attachment, droplet coalescence, and magnetic separation.

The concentration–response curve approached an apparent plateau of approximately 420 mg·g^−1^ under the tested batch conditions. This value is used as an operational capacity metric, not as a molecular monolayer capacity. Surface hydroxyl and extract-derived sulfur/oxygen groups may provide non-covalent contact sites for polar components at oil–droplet interfaces [11,25,26,29,30], while the Fe_3_O_4_ core enables collection of particle–droplet aggregates by an external magnetic field [28,29].

Accordingly, the concentration-dependent data quantify the amount of emulsified oil removed per unit mass of material under the specified batch protocol. The underlying mechanism is discussed as a combined process of interfacial adhesion, droplet association, coalescence, and demulsification-assisted magnetic separation rather than as strict adsorption equilibrium.

### 2.4. Robustness of Gel-Related Colloidal Interactions Under Complex Conditions

**Figure 4:**
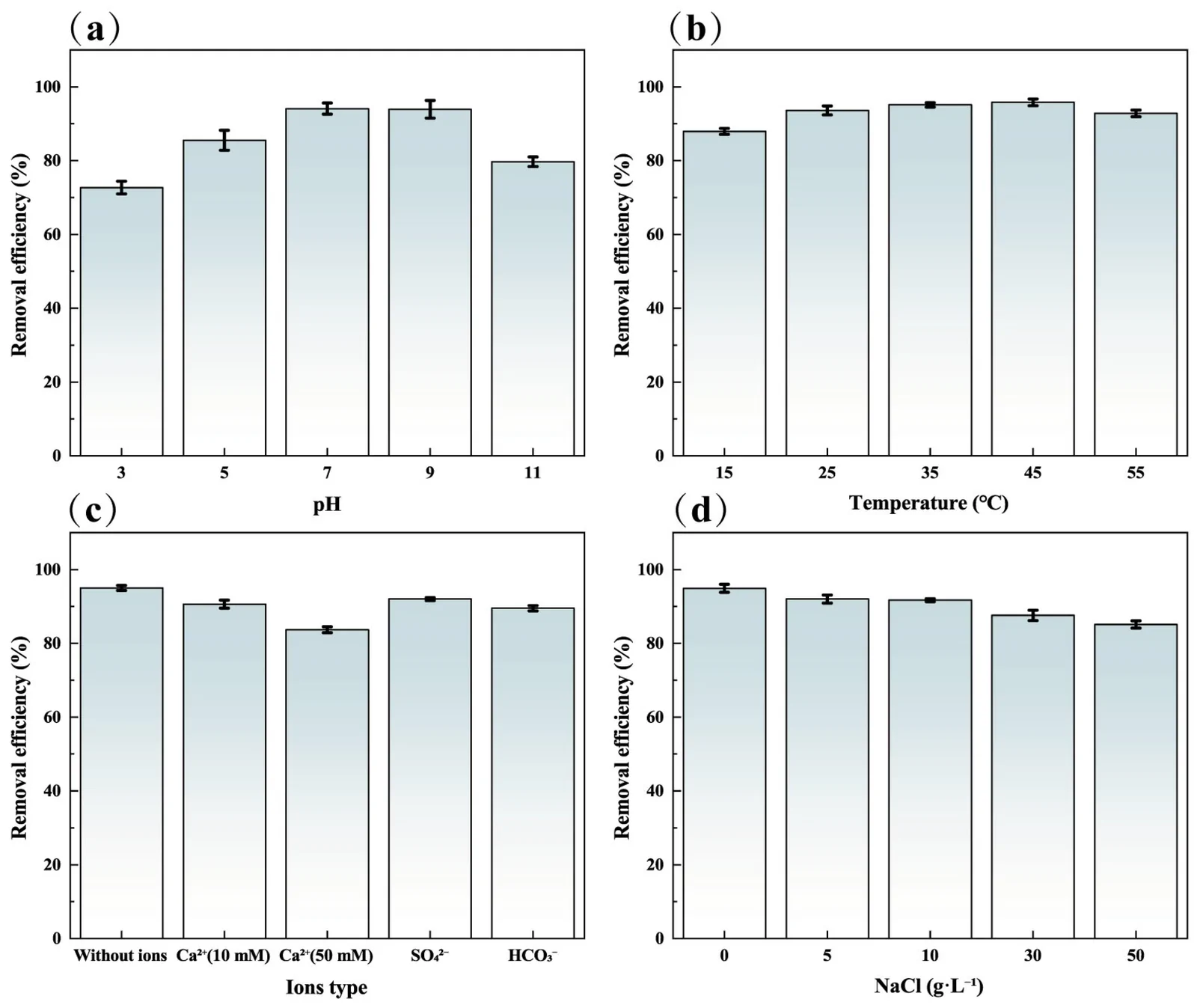
Effects of environmental factors on emulsified oil removal by Allicin@Fe_3_O_4_: (**a**) pH, (**b**) temperature, (**c**) coexisting ions, and (**d**) salinity (NaCl concentration). Bars show mean ± standard deviation from three independently prepared batch experiments (*n* = 3).

To evaluate the robustness of Allicin@Fe_3_O_4_ under complex aqueous conditions, its oil removal performance was examined under different pH, temperature, ionic composition, and salinity conditions (Figure 4). These factors directly influence the surface charge state of particles and oil droplets, the strength of oil–water interfacial films, and the probability of particle–droplet collision and attachment.

Solution pH is an important factor affecting surface charge and interfacial interaction. Different pH conditions can change the dissociation state of surface functional groups and the charge of oil droplets, thereby influencing electrostatic interaction and interfacial capture behavior [31,32]. In this study, Allicin@Fe_3_O_4_ maintained an approximate removal efficiency of 70–95% across pH 3–11 and achieved its best performance under neutral-to-mildly alkaline conditions (Figure 4a). Removal decreased at the strongly acidic and alkaline limits, indicating that the extract-derived surface layer retains interfacial activity over the tested range but remains sensitive to protonation state and particle/droplet charge.

Across 15–55 °C, the mean removal efficiencies shown in Figure 4b remained within approximately 88–96%, corresponding to an absolute peak-to-trough spread of about 8 percentage points. We therefore describe the temperature response as modest over the tested interval rather than claiming temperature independence or statistical equivalence [33,34]. High inorganic salt concentrations can compress the electrical double layer around oil droplets and alter the interfacial film [35,36]. Under the tested ion types and NaCl concentrations of 5–50 g·L^−1^, mean removal remained approximately 85–95% (Figure 4c,d), although the descriptive decreases at the most severe conditions show that ionic competition and ionic strength were not negligible.

These results show that the interfacial activity of Allicin@Fe_3_O_4_ persists under the tested pH, temperature, ion, and salinity conditions, although the pH extremes and high salinity produce measurable performance losses. In the proposed gel-like framework, electrolyte composition and protonation state regulate the particle–droplet interactions that precede magnetic collection. Together with the recoverability of the Fe_3_O_4_ core [37], the results support further evaluation in complex emulsified oily wastewater rather than establishing full-scale suitability; real wastewater and continuous flow validation remain necessary.

### 2.5. Magnetic Recovery and Cyclic Interfacial Activity

**Figure 5:**
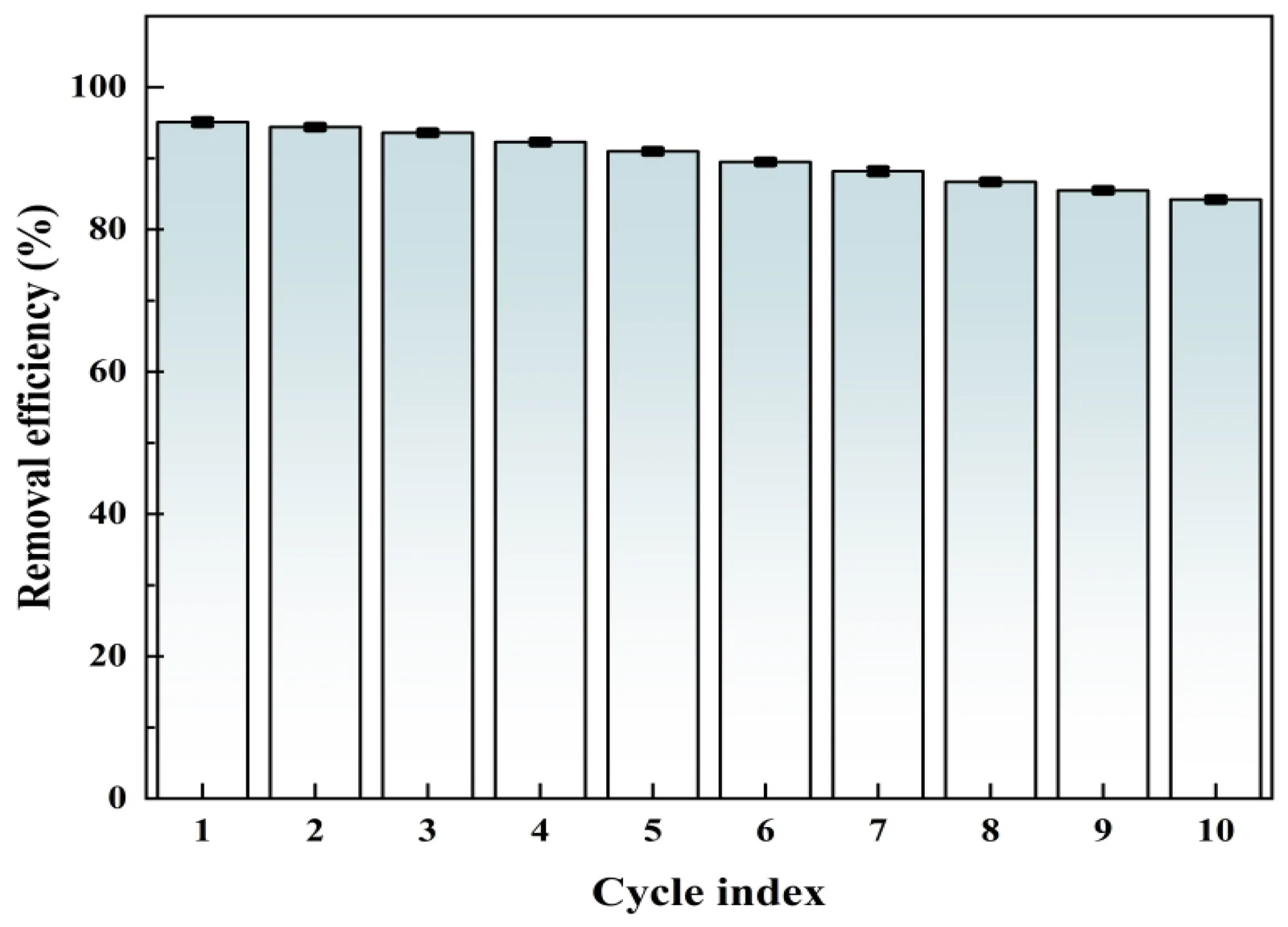
Reusability of Allicin@Fe_3_O_4_ over ten capture–regeneration cycles. Bars show mean ± standard deviation from three independently prepared batch experiments (*n* = 3).

The magnetic recovery and cyclic stability of Allicin@Fe_3_O_4_ were evaluated through repeated oil capture–regeneration experiments [38]. As shown in Figure 5, the removal efficiency gradually decreased from approximately 95% in the first cycle to approximately 84% after ten cycles, corresponding to an overall decline of less than 12%. During the first five cycles, the removal efficiency remained above 90%, indicating that the extract-derived interfacial functional layer retained most of its oil–droplet capture ability during repeated use.

The slight performance decay after extended cycling may be related to irreversible occupation of some interfacial sites, partial loss or deactivation of surface functional groups, and residual oil blocking particle surfaces [39]. Similar performance attenuation has been reported for other magnetic nanoadsorbent systems, where surface fouling or partial desorption of the organic functional layer can reduce the number of effective capture sites [40].

The Fe_3_O_4_ core enabled prompt collection with the permanent magnet used in the batch protocol, reducing material loss during solid–liquid separation and supporting repeated operation. The magnet’s surface field, field gradient, separation distance, and collection time were not quantified; the present results therefore demonstrate qualitative magnetic recoverability but do not define a minimum field required for deactivation or scale-up. These engineering parameters should be measured in future continuous-flow studies.

### 2.6. Representative Molecular Motif for Organosulfur-Mediated Interfacial Anchoring

**Figure 6:**
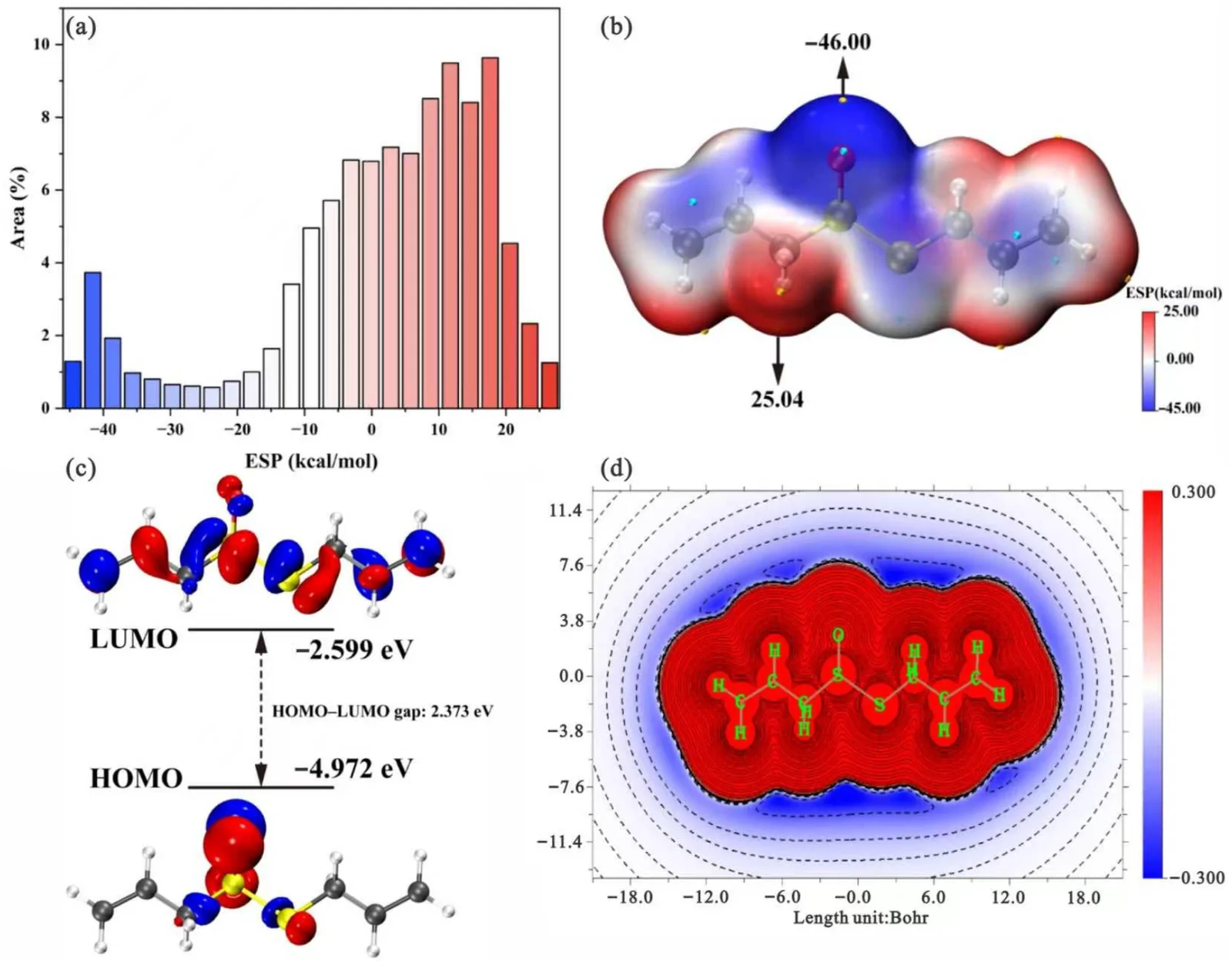
Molecular descriptors calculated for allicin as a representative garlic organosulfur molecule: (**a**,**b**) electrostatic potential distribution; (**c**) frontier molecular orbitals and energy levels; and (**d**) two-dimensional van der Waals potential map. These calculations illustrate a plausible interaction motif and do not establish the composition of the extract-derived coating.

To connect the macroscopic removal behavior with one plausible molecular interaction motif, electrostatic potential (ESP), frontier molecular orbitals (FMO), and two-dimensional van der Waals (vdW) potential were calculated for allicin as a representative garlic organosulfur molecule (Figure 6). These calculations do not determine the composition of the extract-derived particle surface. Instead, they show how an allicin-like thiosulfinate structure could provide a heterogeneous electronic surface in which the sulfoxide/sulfur region offers polar contact sites and the allyl segments offer weakly hydrophobic orientation toward the oil phase.

The ESP distribution shows a strongly heterogeneous molecular surface, with the most negative potential (−46.00 kcal·mol^−1^) localized around the sulfoxide oxygen (S=O) and the positive region (25.04 kcal·mol^−1^) distributed mainly along the allyl carbon–hydrogen chain and the area adjacent to sulfur (Figure 6a,b) [41,42]. This coexistence of electron-rich and electron-deficient regions provides the molecular basis for directional interaction at oil–water interfaces. In emulsified oily wastewater, the S=O-centered polar region can interact with water molecules and polar oil components, such as heteroatomic groups in asphaltenes and resins, through electrostatic interaction and hydrogen bonding, while the allyl segments tend to orient toward the oil phase [43,44].

FMO analysis provides further electronic-structure support for this interfacial role. The HOMO and LUMO energies of allicin are −4.972 and −2.599 eV, respectively, corresponding to a HOMO-LUMO gap of 2.373 eV (Figure 6c). This relatively small gap indicates that allicin can undergo charge redistribution and polarization when exposed to polar interfaces or when interacting with surface-active oil components [45]. The donor-acceptor character implied by the HOMO/LUMO distribution favors polarization coupling with asphaltene- and resin-like species enriched at the oil–droplet surface, thereby weakening the original interfacial film, promoting droplet coalescence, and increasing the adhesion tendency between oil droplets and Allicin@Fe_3_O_4_ particles [46].

The 2D vdW potential map likewise indicates an anisotropic distribution of non-covalent interaction regions on the modeled allicin molecule (Figure 6d). Attractive regions around sulfur and the adjacent sulfoxide group could favor contact with polar oil components, whereas hydrocarbon segments could orient toward the oil phase [47,48,49]. This calculation supports a plausible interaction motif for an allicin-containing extract-derived coating but cannot establish that allicin is predominant on Fe_3_O_4_. The experimentally observed demulsification-assisted separation is therefore interpreted using the combined evidence from surface characterization, batch capture, and magnetic recovery rather than from the molecular calculation alone [50].

### 2.7. Integrated Mechanism of Gel-like Interfacial Capture and Magnetic Separation

Based on the experimental and molecular-level results, oil removal by Allicin@Fe_3_O_4_ is interpreted through a coupled sequence of interfacial attachment, droplet association, coalescence, and magnetic separation [28,29,38]. The garlic-extract-derived organic layer provides mixed polar and weakly hydrophobic functionalities, while Fe_3_O_4_ supplies a recoverable magnetic core. This organic layer–magnetic core architecture may allow particles to bridge or associate with more than one emulsified droplet, consistent with interfacially active magnetic particle systems reported in the literature [25,28,29]. The term gel-like describes transient particle–droplet structuring and does not imply chemically crosslinked or rheologically confirmed bulk gelation.

First, the polar groups on allicin can interact with heteroatom-containing components in oil droplets, whereas the hydrophobic segments orient toward the oil phase. This amphiphilic-like arrangement helps reduce local interfacial stability and enhances particle attachment at the droplet surface [26,51]. Second, the attached particles can disrupt the protective interfacial film formed by natural surface-active substances, promoting droplet coalescence and growth [29]. Third, sulfur-containing groups can selectively interact with polar aromatic components such as asphaltenes and resins, further weakening the stability of emulsified droplets [30,52,53].

Finally, once the oil droplets are anchored and partially coalesced, the Fe_3_O_4_ core enables rapid magnetic separation, forming a sequence of interfacial anchoring, gel-like particle–droplet association, droplet coalescence, and magnetic recovery [54]. Allicin@Fe_3_O_4_ therefore functions as an interfacially active magnetic-colloid building block rather than merely as a conventional adsorbent. This multiscale interpretation links bio-derived surface functionalization to a gel-related environmental application while retaining the evidential boundary that bulk viscoelastic gelation was not directly measured.

Bio-derived origin should not be equated with environmental neutrality. Garlic extract contains multiple organic and organosulfur constituents, and their release, transformation, and ecotoxicity were not quantified in this study. Repeated water/ethanol washing was used to remove unbound organics during synthesis, and organic-containing washings and regeneration eluates require controlled waste handling. Before environmental application, total organic carbon release, leaching/speciation, acute and chronic aquatic toxicity, and life-cycle impacts should be evaluated using real wastewater.

## 3. Conclusions

This study developed garlic-extract-functionalized Allicin@Fe_3_O_4_ magnetic colloids as organic–inorganic building blocks for gel-like interfacial capture and magnetic separation of emulsified oil. The sample name is operational: FTIR and XRD support an extract-derived organic sulfur/oxygen-containing layer and crystalline Fe_3_O_4_, but they do not prove that allicin is the predominant surface species. Under the tested batch conditions, more than 95% of emulsified oil was removed within 20 min, the experimental equilibrium apparent uptake was approximately 380 mg·g^−1^, and removal remained above 90% after five cycles and approximately 84% after ten cycles. The concentration–response data are treated as apparent capture capacity rather than a strict adsorption isotherm, and temperature effects are discussed descriptively rather than through a van’t Hoff equilibrium analysis. The primary particles observed by SEM were nanoscale with larger aggregated domains, but quantitative particle-size distributions, field-strength thresholds, real-wastewater ecotoxicity, continuous-flow performance, and rheological validation remain necessary.

## 4. Materials and Methods

### 4.1. Materials

Fresh garlic (*Allium sativum*) was purchased from a local farmers’ market, peeled, and cleaned before use. Iron(III) chloride hexahydrate (FeCl_3_·6H_2_O, analytical grade), iron(II) chloride tetrahydrate (FeCl_2_·4H_2_O, analytical grade), ammonia solution (25 wt%), anhydrous ethanol (analytical grade), n-hexane (analytical grade), hydrochloric acid (HCl, analytical grade), and sodium hydroxide (NaOH, analytical grade) were all purchased from Sinopharm Chemical Reagent Co., Ltd. (Shanghai, China). Sodium chloride (NaCl), sodium sulfate (Na_2_SO_4_), sodium bicarbonate (NaHCO_3_), calcium chloride (CaCl_2_), and magnesium chloride (MgCl_2_), all of analytical grade, were also purchased from Sinopharm Chemical Reagent Co., Ltd. (Shanghai, China) and were used to simulate various salinity and coexisting ion conditions. Sodium dodecyl sulfate (SDS, analytical grade, Sinopharm Chemical Reagent Co., Ltd., Shanghai, China) was used to prepare simulated emulsified oily wastewater. Crude oil samples were obtained from Xinjiang Oilfield and subjected to simple filtration to remove impurities before use. Deionized water (resistivity 18.2 MΩ·cm), prepared by a laboratory ultrapure water system, was used for all solution preparations.

### 4.2. Preparation of Garlic Extract

Fresh garlic cloves (*Allium sativum*) were peeled, quickly rinsed with deionized water, and surface-dried. Garlic cloves (50.0 g) were weighed, chopped, and placed into a pre-cooled grinding cup. Then, 100 mL of 70% (*v*/*v*) ethanol was added (solid-to-liquid ratio of 1:2, g:mL), and the mixture was homogenized at high speed for 1–2 min in an ice bath using a homogenizer (T18 digital ULTRA-TURRAX, IKA-Werke GmbH & Co. KG, Staufen, Germany) to obtain a uniform slurry [55]. Subsequently, the slurry was allowed to stand in the dark at room temperature (20–25 °C) for 10–15 min to promote the garlic enzyme-catalyzed reaction and generate bioactive sulfur-containing components such as allicin. After standing, the mixture was magnetically stirred for 30 min at 4 °C (protected from light throughout the extraction process). Following extraction, the mixture was roughly filtered through double-layer gauze to remove large residues. The resulting filtrate was centrifuged at 8000 rpm for 10 min at 4 °C. The supernatant was collected and further filtered through a 0.45 μm microporous membrane (Tianjin Jinteng Experimental Equipment Co., Ltd., Tianjin, China) to obtain the final garlic extract (GE). The obtained GE was aliquoted into brown centrifuge tubes (Corning Inc., Corning, NY, USA), stored at 4 °C protected from light, and used within 48 h to minimize degradation of active components.

### 4.3. Synthesis of Allicin@Fe_3_O_4_ Magnetic Colloids

Under nitrogen protection, FeCl3·6H2O (5.40 g, 20 mmol) and FeCl2·4H2O (1.99 g, 10 mmol) were dissolved in 100 mL of deoxygenated deionized water. After dissolution at 80 °C and 600 rpm, 10 mL of garlic extract was added and stirred for 10 min. Ammonia solution (25 wt%) was then added dropwise to pH 10.0 ± 0.2, and the reaction proceeded for 60 min at 80 °C under nitrogen. The black product was magnetically separated and washed three times with deionized water and ethanol to remove residual ions and unbound extract-derived organic compounds. Washings were collected as organic-containing laboratory waste rather than discharged. The product was vacuum-dried at 50–60 °C for 12 h and stored at 4 °C in brown vials. Because the extract contains multiple labile organosulfur species, the product name Allicin@Fe_3_O_4_ is operational and does not identify a single purified surface ligand [56].

### 4.4. Characterization of Allicin@Fe_3_O_4_ Magnetic Colloids

SEM (Hitachi Regulus 8100, Tokyo, Japan) was used to examine morphology and provide an image-based estimate of primary particle size and aggregation state. FTIR (Thermo Fisher Scientific Nicolet iS20, Waltham, MA, USA) was used to assess Fe–O, hydroxyl, aliphatic, and overlapping sulfur/oxygen-containing surface functionalities; it was not used as species-specific proof of allicin. XRD (PANalytical X’Pert PRO MPD, Almelo, The Netherlands) was used to verify the Fe_3_O_4_ crystalline phase. Quantitative particle-size distributions by TEM/DLS and chemical speciation of the organic coating by chromatography or mass spectrometry were not available and are identified as limitations.

### 4.5. Batch Oil–Droplet Capture Experiment

To evaluate the oil–droplet capture performance of Allicin@Fe_3_O_4_ in oily wastewater, batch interfacial capture experiments were conducted using simulated emulsified oily wastewater. The simulated oily wastewater was prepared by adding a certain amount of crude oil to deionized water, along with a small amount of surfactant (SDS, 0.1 g·L^−1^), and emulsified using an ultrasonic processor (JY92-IIN, Ningbo Scientz Biotechnology Co., Ltd., Ningbo, China) to form a stable oil-in-water (O/W) emulsion. Different initial oil concentrations (C_0_) were obtained by adjusting the amount of oil added. Specifically, 50 mL of the emulsion was placed into a 100 mL conical flask, and a specific mass (m) of Allicin@Fe_3_O_4_ (material dosage) was added. The flask was shaken in a constant-temperature shaker (ZHWY-200B, Shanghai Zhicheng Analytical Instrument Mfg. Co., Ltd., Shanghai, China) at 200 rpm for oil–droplet capture, with the reaction temperature set at 25 °C. Upon reaching a predetermined contact time (t), the material was separated using an external magnetic field, and the supernatant was collected for oil content determination [11].

For oil–droplet capture kinetics, the initial oil concentration and material dosage were fixed and independent samples were collected over 0–120 min [57]. For concentration-response experiments, the initial oil concentration was varied under the selected equilibrium-time condition [58]. Oil content was determined by n-hexane extraction–infrared spectrophotometry against a crude-oil calibration curve [59]. Each condition comprised three independently prepared and processed batch experiments (*n* = 3), rather than three instrumental readings from one sample. A blank control was included in each experimental set, and results are reported as mean ± standard deviation.

### 4.6. Environmental Robustness Experiment

To systematically evaluate the impact of different environmental conditions on the oily wastewater removal performance of Allicin@Fe_3_O_4_, the effects of solution pH, salinity, temperature, and coexisting ions on oil removal efficiency were investigated under batch capture equilibrium conditions (equilibrium time determined from Section 4.5) [60]. Unless otherwise specified, all experiments used the same initial oil concentration, material dosage, solution volume, and shaking conditions. After capture, the material was separated by an external magnetic field, and the oil content in the supernatant was measured to calculate the removal efficiency [61].

#### 4.6.1. pH Influence

The pH of the simulated oily wastewater was adjusted to 3, 5, 7, 9, and 11 using 0.1 M HCl or 0.1 M NaOH. Oil–droplet capture reactions were conducted in a constant-temperature oscillator at 25 °C and 200 rpm. After capture, the material was magnetically separated, and the oil content in the supernatant was measured. By comparing removal efficiencies under different pH conditions, the influence of solution acidity/alkalinity on the material’s surface charge, interfacial capture behavior, and oil–droplet capture efficiency was analyzed [62].

#### 4.6.2. Salinity Influence

To simulate high-salinity produced water environments, NaCl was added to the simulated oily wastewater to create different salinity conditions (0, 5, 10, 30, and 50 g·L^−1^). Oil–droplet capture experiments were conducted under otherwise constant conditions, and the removal efficiencies and apparent uptake capacities at different salinities were determined to evaluate the impact of ionic strength changes on oil droplet stability and capture behavior.

#### 4.6.3. Temperature Influence

The constant-temperature oscillator was set to 15, 25, 35, 45, and 55 °C [63,64]. Oil–droplet capture experiments were performed under identical initial oil concentration, material dosage, solution volume, and contact-time conditions. After magnetic separation, the oil content of the supernatant was measured. These tests were used to describe the sensitivity of removal performance to temperature within the tested batch range; they were not treated as an equilibrium thermodynamic experiment.

#### 4.6.4. Influence of Coexisting Ions

To simulate the complex inorganic salt environment in actual oily wastewater, typical cations (Ca^2+^, Mg^2+^) and anions (SO_4_^2−^, HCO_3_^−^) were separately added to the simulated oily wastewater at concentrations of 1, 10, and 50 mM. Experiments were conducted under identical capture conditions, and the changes in removal efficiency under different coexisting ion conditions were compared to assess the impact of divalent ions and bicarbonate species on the material’s oil removal performance [65].

### 4.7. Magnetic Recovery and Recycling Potential

For each capture–regeneration cycle, the material was collected with the same permanent magnet and regenerated by ethanol elution: the recovered material was ultrasonicated in ethanol for 5–10 min and shaken for 10 min, and washing was repeated two or three times until no visible oil color remained. The organic-containing eluate was retained for controlled solvent-waste disposal. The regenerated material was rinsed with deionized water, vacuum-dried at 50–60 °C for 12 h, and reused. Ten cycles were tested. Magnet surface field, field gradient, separation distance, and collection time were not quantified; consequently, the study evaluates repeatability with a fixed laboratory magnet but does not establish a minimum magnetic field for process deactivation or scale-up.

(1) $$Retention(\%)=\frac{R_{n}}{R_{1}}\times 100\%$$

Here, $R_{1}$ represents the removal efficiency in the first cycle, and $R_{n}$ represents the removal efficiency in the n-th cycle.

### 4.8. Quantum Chemistry Calculation and Analysis

All quantum-chemical calculations were performed using ORCA 6.0.0 [66]. Geometry optimization and frequency calculations for allicin, selected as a representative garlic organosulfur molecule, were conducted at the r2SCAN-3c and B3LYP-D3(BJ)/def2-TZVP levels. The optimized wavefunction was analyzed with Multiwfn (version 3.8) [67] to obtain the electrostatic potential, frontier molecular orbitals, and van der Waals potential. These descriptors were used to illustrate a plausible interaction motif for an allicin-containing extract-derived surface layer; they were not used to determine or quantify the chemical species present on the particle surface.

### 4.9. Statistical Analysis and Reporting

All batch experiments were performed as three independently prepared experimental replicates, and values are reported as mean ± standard deviation (*n* = 3). The present manuscript uses descriptive comparisons of treatment means and does not make claims of statistical significance; accordingly, inferential terms such as ‘significant’ and post hoc significance groupings are not used. Replicate-level data and a prespecified inferential analysis would be required before reporting ANOVA or multiple-comparison *p*-values.

## Data Availability

The raw data supporting the conclusions of this article will be made available by the authors on request.

## References

[1] Abuhasel K., Kchaou M., Alquraish M., Munusamy Y., Jeng Y.T. (2021). Oily Wastewater Treatment: Overview of Conventional and Modern Methods, Challenges, and Future Opportunities. Water.

[2] Wang Y., Zhang Y., Liang L., Tu F., Li Z., Tang X., Dai L., Li L. (2024). Research Progress on Membrane Separation Technology for Oily Wastewater Treatment. Toxics.

[3] Al-Husaini I.S., Al Haddabi M.H. (2025). Recent Advances in Functionalized Electrospun Nanofiber Membranes for Enhanced Oily Water Treatment. J. Environ. Chem. Eng..

[4] Diraki A., Mackey H.R., McKay G., Abdala A. (2019). Removal of Emulsified and Dissolved Diesel Oil from High Salinity Wastewater by Adsorption onto Graphene Oxide. J. Environ. Chem. Eng..

[5] Li Z., Chen W., Yang F., Wang Y., Cui S., Li Q., Nie Z., Wei Q. (2025). Porous Ceramic Membranes Fabricated Mainly from Red Mud for Oily Wastewater Treatment. Sep. Purif. Technol..

[6] Ouyang E., Hu Z., Xu S., Yang W., Yang H., Li K., Zhao R. (2026). Efficient Demulsification of Emulsified Oil Wastewater by Graft-Functionalized *β*-Cyclodextrin Flocculants: The Effect of Charge Density, Hydrophobicity, and Graft Chain Length. Water Res..

[7] Medeiros A.D.M.d., Silva Junior C.J.G.d., Amorim J.D.P.d., Durval I.J.B., Costa A.F.d.S., Sarubbo L.A. (2025). Oily Wastewater Treatment: Methods, Challenges, and Trends. Processes.

[8] Nguyen M.D., Tran H.-V., Xu S., Lee T.R. (2019). Fe_3_O_4_ Nanoparticles: Structures, Synthesis, Magnetic Properties, Surface Functionalization, and Emerging Applications. Appl. Sci..

[9] Liu S., Yu B., Wang S., Shen Y., Cong H. (2020). Preparation, Surface Functionalization and Application of Fe_3_O_4_ Magnetic Nanoparticles. Adv. Colloid Interface Sci..

[10] Xu J., Chen Y., Luo J., Xu J., Zhou G., Yu Y., Xue L., Yang L., He S. (2022). Fe_3_O_4_ Nanoparticles Affect Paddy Soil Microbial-Driven Carbon and Nitrogen Processes: Roles of Surface Coating and Soil Types. Environ. Sci. Nano.

[11] Hamedi H., Zendehboudi S., Rezaei N., Azizi A., Shahhoseini F. (2023). Application of Functionalized Fe_3_O_4_ Magnetic Nanoparticles Using CTAB and SDS for Oil Separation from Oil-in-Water Nanoemulsions. Langmuir.

[12] Imsong R., Ahmed F.U., Dhar Purkayastha D. (2025). Tailoring MXene-based Electrospun Membranes with Spindle-Like Structures for Multifunctional Oily Wastewater Treatment. Chem. Eng. J..

[13] Borlinghaus J., Albrecht F., Gruhlke M., Nwachukwu I., Slusarenko A. (2020). Allicin: Chemistry and Biological Properties. Molecules.

[14] Chauhan A., Dewali S., Pathak V.M., Bisht S.S., Chauhan R., Kaur D., Tuli H.S., Haque S., Ahmad F. (2025). Therapeutic Role of Allicin in Gastrointestinal Cancers: Mechanisms and Safety Aspects. Discov. Oncol..

[15] Hu L., Zhao P., Wei Y., Lei Y., Guo X., Deng X., Zhang J. (2024). Preparation and Characterization Study of Zein–Sodium Caseinate Nanoparticle Delivery Systems Loaded with Allicin. Foods.

[16] Hernández-Hernández A.A., Cariño-Cortes R., Mendoza-Huizar L.H., Aguirre-Álvarez G., Rivera M., Jiménez-Alvarado R. (2025). Synthesis and Characterization of Core–Shell Nanoparticles (Fe_3_O_4_@TEOS) Biofunctionalized with Allicin from Allium Sativum Optimized by a Box–Behnken Design. Chem. Pap..

[17] Hur J.U., Choi J.S., Choi S.-C., An G.S. (2020). Highly Dispersible Fe_3_O_4_ Nanoparticles via Anionic Surface Modification. Polymers.

[18] Boily J.-F., Felmy A.R. (2008). On the Protonation of Oxo- and Hydroxo-Groups of the Goethite (*α*-FeOOH) Surface: A FTIR Spectroscopic Investigation of Surface O–H Stretching Vibrations. J. Water Process Eng..

[19] Ebadi M., Rifqi Md Zain A., Tengku Abdul Aziz T.H., Mohammadi H., Tee C.A.T., Rahimi Yusop M. (2023). Formulation and Characterization of Fe_3_O_4_@PEG Nanoparticles Loaded Sorafenib; Molecular Studies and Evaluation of Cytotoxicity in Liver Cancer Cell Lines. Polymers.

[20] Husain S., Irfansyah M., Haryanti N.H., Suryajaya S., Arjo S., Maddu A. (2019). Synthesis and Characterization of Fe_3_O_4_ Magnetic Nanoparticles from Iron Ore. J. Phys. Conf. Ser..

[21] Salman D., Juzsakova T., Al-Mayyahi M.A., Ákos R., Mohsen S., Ibrahim R.I., Mohammed H.D., Abdullah T.A., Domokos E., Korim T. (2021). Synthesis, Surface Modification and Characterization of Magnetic Fe_3_O_4_@SiO_2_ Core-Shell Nanoparticles. J. Phys. Conf. Ser..

[22] Serrano M., Lira-Diaz E., Márquez-Aguilar P.A., Acevedo-Fernández J.J., Parra-Parra A. (2024). In Vitro Cell Viability Evaluation of Amorphous Carbon and Fe_3_O_4_ Compounds. MRS Adv..

[23] Chen Y., Su R., Xu F., Ma M., Wang Y., Ma D., Li Q. (2024). Oxygen-Containing Functional Groups in Fe_3_O_4_@three-Dimensional Graphene Nanocomposites for Enhancing H_2_O_2_ Production and Orientation to ^1^O_2_ in Electro-Fenton. J. Hazard. Mater..

[24] Hamedi H., Rezaei N., Zendehboudi S. (2022). Investigation of Emulsified Oil Adsorption onto Functionalized Magnetic Nanoparticles—Kinetic and Isotherm Models. Energies.

[25] Dickhout J.M., Kleijn J.M., Lammertink R.G.H., de Vos W.M. (2018). Adhesion of Emulsified Oil Droplets to Hydrophilic and Hydrophobic Surfaces—Effect of Surfactant Charge, Surfactant Concentration and Ionic Strength. Soft Matter.

[26] Wu W., Ma X., Wang R., Hao M. (2026). The Effect of Electrostatic Interaction and Wettability of Fe3O4 Nano-Composites on Demulsification of O/W (Hexadecane) Nanoemulsion. Water Sci. Technol..

[27] Jia T., Niu L., Qi Z., Cong W., Xi J., Yang C., Peng Y. (2025). The Biosynthesis, Storage and Utilization of Elemental Sulfur: Enzymatic Pathways, Molecular Mechanisms, and Future Perspectives. Crit. Rev. Environ. Sci. Technol..

[28] Bai Y., Kong Y., Yang M., Wang X., Sun D., Zhou J. (2023). Magnetic Composite Lignin Colloids Prepared with a Convenient Patching Method for Generally Treating Heterogeneous Oily Wastewater. Part. Part. Syst. Charact..

[29] Elmobarak W.F., Almomani F. (2021). Application of Fe3O4 Magnetite Nanoparticles Grafted in Silica (SiO2) for Oil Recovery from Oil in Water Emulsions. Chemosphere.

[30] Lian Q., Islam F., Ahmad Z.U., Lei X., Depan D., Zappi M., Gang D.D., Holmes W., Yan H. (2021). Enhanced Adsorption of Resorcinol onto Phosphate Functionalized Graphene Oxide Synthesized via Arbuzov Reaction: A Proposed Mechanism of Hydrogen Bonding and *π*-*π* Interactions. Geochim. Cosmochim. Acta.

[31] Cherukupally P., Sun W., Wong A.P.Y., Williams D.R., Ozin G.A., Bilton A.M., Park C.B. (2019). Surface-Engineered Sponges for Recovery of Crude Oil Microdroplets from Wastewater. Nat. Sustain..

[32] Hashemi-Moghaddam H. (2024). Role of Magnetic Polymer–Coated Nanoparticles in Separation Sciences. Magnetic Nanoparticles and Polymer Nanocomposites.

[33] Al-Ghouti M.A., Al-Absi R.S. (2020). Mechanistic Understanding of the Adsorption and Thermodynamic Aspects of Cationic Methylene Blue Dye onto Cellulosic Olive Stones Biomass from Wastewater. Sci. Rep..

[34] Aumeier B.M., Augustin A., Thönes M., Sablotny J., Wintgens T., Wessling M. (2022). Linking the Effect of Temperature on Adsorption from Aqueous Solution with Solute Dissociation. J. Hazard. Mater..

[35] Miadonye A., Amadu M. (2023). Theoretical Interpretation of pH and Salinity Effect on Oil-in-Water Emulsion Stability Based on Interfacial Chemistry and Implications for Produced Water Demulsification. Processes.

[36] Shi R., Ow H., Cox J.R., Kmetz A.A., Chen H. (2022). Optimizing Colloidal Stability and Transport of Polysaccharide-Coated Magnetic Nanoparticles for Reservoir Management: Effects of Ion Specificity. Processes.

[37] Simonsen G., Strand M., Øye G. (2018). Potential Applications of Magnetic Nanoparticles Within Separation in the Petroleum Industry. J. Pet. Sci. Eng..

[38] Flores J.A., Pavía-Sanders A., Chen Y., Pochan D.J., Wooley K.L. (2015). Recyclable Hybrid Inorganic/Organic Magnetically Active Networks for the Sequestration of Crude Oil from Aqueous Environments. Chem. Mater..

[39] Wei S., Liu Y., Rezaei A., Ege D., Kamali A.R. (2024). Circular Economy Approach for Thermal Regeneration of Graphene-Encapsulated Magnetic Nanoparticles as an Efficient Adsorbent for Sustainable Wastewater Management. J. Alloys Compd..

[40] Abolghasemi S., Nasiri A., Hashemi M., Rajabi S., Rahimi F. (2025). Magnetic Nanocomposites: Innovative Adsorbents for Antibiotics Removal from Aqueous Environments–a Narrative Review. J. Alloys Compd..

[41] Wei D., Feng Y., Guo Y., Su Y., Huang K., Lan L., Li H., Zhang J., Lan P., Tang L. (2025). Adsorption of Methylene Blue on Starch Sulfate: Insights from Density Functional Theory. Appl. Water Sci..

[42] Zhang Y., Zhang S., Xu S., Cao F., Ren X., Sun Q., Yang L., Wennersten R., Mei N. (2024). Simulation of the VOC Adsorption Mechanism on Activated Carbon Surface by Nitrogen-Containing Functional Groups. Appl. Sci..

[43] Song S., Li C., Jia W., Maeda N., Zhang X., Xiao H., Yang F., Zhang Y. (2025). Adsorption Behavior of Non-Ionic Demulsifiers at the Oil/Water Interface Stabilized by Asphaltenes: Experiments, Adsorption Kinetics, and Mechanisms. Sep. Purif. Technol..

[44] Liu J., Zhao Y., Ren S. (2015). Molecular Dynamics Simulation of Self-Aggregation of Asphaltenes at an Oil/Water Interface: Formation and Destruction of the Asphaltene Protective Film. Geochim. Cosmochim. Acta.

[45] Murray J.S., Politzer P. (2011). The Electrostatic Potential: An Overview. WIREs Comput. Mol. Sci..

[46] Lu T., Chen F. (2012). Multiwfn: A Multifunctional Wavefunction Analyzer. J. Comput. Chem..

[47] Xie Z., Liu S. (2025). Interface Mechanism of Promoting Low-Rank Coal Flotation by Characteristic Groups in Hydrophilic Moieties of Cationic–Anionic Surfactants. Sep. Purif. Technol..

[48] Li M., Acero A.A., Huang Z., Rice S.A. (1994). Formation of an Ordered Langmuir Monolayer by a Non-Polar Chain Molecule. Chemosphere.

[49] Zhang X., Cao Y., Yang D., Yang W., Yan B., Zhang L. (2025). Thermally Regulated Flocculation-Coalescence Process by Temperature-Responsive Cationic Polymeric Surfactant for Enhanced Crude Oil-Water Separation. Water Sci. Technol..

[50] Hassan M., Naidu R., Du J., Liu Y., Qi F. (2020). Critical Review of Magnetic Biosorbents: Their Preparation, Application, and Regeneration for Wastewater Treatment. Sci. Total Environ..

[51] Hashemi S., Saien J. (2019). Equilibrium and Dynamic Interfacial Tensions of Oil/Water in the Presence of an Imidazolium Ionic Liquid Strengthen with Magnetite Nanoparticles. J. Mol. Liq..

[52] Yang Y., Jin H., Chen B., Zhang Y., Cai Z., Sheng L. (2025). Disturbing Egg Yolk Protein Structure via pH-shifting Treatment for Interface Reorganization: Improving Solubility to Enhance Oil-Water Interface Adsorption and Emulsification Properties. Energies.

[53] Wang S., Yu Y., He L., Zhang D., Ye M. (2020). Design of Magnetic Nanoparticles with High Magnetic Separation Efficiencies and Durability for Cu^2+^ Adsorption. Nanotechnology.

[54] Hojatjalali M., Bahraminia S., Anbia M. (2025). Superhydrophobic Magnetic Melamine Sponge Modified by Flowerlike ZnO and Stearic Acid Using Dip Coating Method for Oil and Water Separation. Sci. Rep..

[55] Chen K.-Q., Lei H.-B., Liu X., Cao W.-J. (2025). Mini-Review: The Health Benefits and Applications of Allicin. Front. Pharmacol..

[56] Dudchenko N., Pawar S., Perelshtein I., Fixler D. (2022). Magnetite Nanoparticles: Synthesis and Applications in Optics and Nanophotonics. Materials.

[57] Zahakifar F., Khanramaki F. (2024). Continuous Removal of Thorium from Aqueous Solution Using Functionalized Graphene Oxide: Study of Adsorption Kinetics in Batch System and Fixed Bed Column. Sci. Rep..

[58] Alatabe M.J.A., Faris H.A., Husham H. (2023). Natural Biosorbent for Oil Adsorption from Produced Water by Sedge Cane. J. Ecol. Eng..

[59] Nur A., Hajrah H., Ilyas A., Aisyah A., Escalante M.C.L. (2022). Determination and Characterization of Lycopene Compounds from N-Hexane Fraction of Tomato Fruit (Solanum Lycopersocum Grandyfolium) by Using Fourier Transform Infrared and UV-Vis Spectrophotometry. Walisongo J. Chem..

[60] Mirshahghassemi S., Cai B., Lead J.R. (2016). Evaluation of Polymer-Coated Magnetic Nanoparticles for Oil Separation Under Environmentally Relevant Conditions: Effect of Ionic Strength and Natural Organic Macromolecules. Environ. Sci. Nano.

[61] Diraki A., Mackey H.R., McKay G., Abdala A. (2022). Removal and Recovery of Dissolved Oil from High-Salinity Wastewater Using Graphene–Iron Oxide Nanocomposites. Appl. Sci..

[62] Álvarez-Esmorís C., Rodríguez-López L., Núñez-Delgado A., Álvarez-Rodríguez E., Fernández-Calviño D., Arias-Estévez M. (2022). Influence of pH on the Adsorption-Desorption of Doxycycline, Enrofloxacin, and Sulfamethoxypyridazine in Soils with Variable Surface Charge. Environ. Res..

[63] Zhang Y., Hu A., Xiong P., Zhang H., Liu Z. (2019). Experimental Study of Temperature Effect on Methane Adsorption Dynamic and Isotherm. J. Korean Ceram. Soc..

[64] Singh S., Anil A.G., Naik T.S.S.K., U B., Khasnabis S., Nath B., Kumar V., Subramanian S., Singh J., Ramamurthy P.C. (2022). Mechanism and Kinetics of Cr(VI) Adsorption on Biochar Derived from Citrobacter Freundii Under Different Pyrolysis Temperatures. J. Water Process Eng..

[65] Luo Y., Fu K., Wang D., Luo J. (2025). Nanobubble-Driven Interfacial Interactions of Carbon-Based Adsorbents with Legacy PFAS: Impact of Concentration, pH, and Coexisting Ions. Environ. Sci. Technol..

[66] Neese F., Wennmohs F., Becker U., Riplinger C. (2020). The ORCA Quantum Chemistry Program Package. J. Chem. Phys..

[67] Lu T. (2024). A Comprehensive Electron Wavefunction Analysis Toolbox for Chemists. Multiwfn. J. Chem. Phys..

